# PDGF is Required for Remyelination-Promoting IgM Stimulation of Oligodendrocyte Progenitor Cell Proliferation

**DOI:** 10.1371/journal.pone.0055149

**Published:** 2013-02-01

**Authors:** Jens O. Watzlawik, Arthur E. Warrington, Moses Rodriguez

**Affiliations:** Departments of Neurology and Immunology, Mayo Clinic College of Medicine, Rochester, Minnesota, United States of America; Hannover Medical School, Germany

## Abstract

**Background:**

Promotion of remyelination is a major goal in treating demyelinating diseases such as *multiple sclerosis* (MS). The recombinant human monoclonal IgM, rHIgM22, targets myelin and oligodendrocytes (OLs) and promotes remyelination in animal models of MS. It is unclear whether rHIgM22-mediated stimulation of lesion repair is due to promotion of oligodendrocyte progenitor cell (OPC) proliferation and survival, OPC differentiation into myelinating OLs or protection of mature OLs. It is also unknown whether astrocytes or microglia play a functional role in IgM-mediated lesion repair.

**Methods:**

We assessed the effect of rHIgM22 on cell proliferation in mixed CNS glial and OPC cultures by tritiated-thymidine uptake and by double-label immunocytochemistry using the proliferation marker, Ki-67. Antibody-mediated signaling events, OPC differentiation and OPC survival were investigated and quantified by Western blots.

**Results:**

rHIgM22 stimulates OPC proliferation in mixed glial cultures but not in purified OPCs. There is no proliferative response in astrocytes or microglia. rHIgM22 activates PDGFαR in OPCs in mixed glial cultures. Blocking PDGFR-kinase inhibits rHIgM22-mediated OPC proliferation in mixed glia. We confirm in isolated OPCs that rHIgM22-mediated anti-apoptotic signaling and inhibition of OPC differentiation requires PDGF and FGF-2. We observed no IgM-mediated effect in mature OLs in the absence of PDGF and FGF-2.

**Conclusion:**

Stimulation of OPC proliferation by rHIgM22 depends on co-stimulatory astrocytic and/or microglial factors. We demonstrate that rHIgM22-mediated activation of PDGFαR is required for stimulation of OPC proliferation. We propose that rHIgM22 lowers the PDGF threshold required for OPC proliferation and protection, which can result in remyelination of CNS lesions.

## Introduction

Multiple sclerosis (MS) is a chronic inflammatory demyelinating disease. MS lesions are characterized by myelin loss, infiltration with microglia/macrophages and lymphocytes and increased deposition of astrocytic protein, but not astrocytic proliferation, leading to scar formation. Despite recent advances in anti-inflammatory and immune modulatory therapy, most treatments fail to prevent disease progression. Stimulation of repair is a major goal in MS and other demyelinating diseases. Attempts to enhance repair can be separated into exogenous therapies that transplant cells [1]–[8] and endogenous therapies that stimulate resident cells. Enhancing endogenous remyelination is an attractive approach because oligodendrocytes capable of myelination are abundant throughout the adult brain. Novel reagents under development include high affinity Abs and fragments against LINGO-1, and remyelination promoting antibodies of the IgM isotype. Lingo-1 is a component of the Nogo-66 receptor/p75-signaling complex [9], [10]. LINGO-1 antagonists promote OPC differentiation and myelination *in vitro* and accelerate remyelination *in vivo* after lysolecithin- or cuprizone-induced demyelination [11] and modulate a rat EAE model [12].

Remyelination promoting IgMs are germline gene-encoded natural autoantibodies that target cell surface antigens of OLs and myelin. They promote remyelination in the Theiler’s murine encephalomyelitis virus (TMEV) and lysolecithin-mediated demyelination models of MS [13]–[19]. A study by *Paz Soldan* [20] reported that remyelination promoting IgMs–three mouse and four human–all induced an immediate, transient Ca^2+^-influx in astrocytes (GFAP+) and a delayed, transient Ca^2+^-influx in early (A2B5+, O4−) and late OPCs (A2B5+, O4+) and immature OLs (O4+, O1−) [20] to suggest an IgM-mediated co-activation of astrocytes. The IgM-stimulated Ca^2+^-influxes into astrocytes and oligodendrocytes do not, however, depend on each other [20].

The recombinant human remyelination promoting IgM, rHIgM22, promotes remyelination *in vivo* [15], [18], [19] comparable to the degree induced by the mouse IgM O4 [21] and polyclonal human IgM [18]. Polyclonal human IgM [22], O4 [23] and rHIgM22 [24] inhibit the differentiation of OPCs to mature (MBP+, MOG+) OLs *in vitro*. rHIgM22 also inhibits apoptotic signaling in OPCs [24]. The signaling complex responsible for the anti-apoptotic effect consists of platelet-derived growth factor receptor alpha (PDGFαR), integrin αvβ3 and the Src family kinase (SFK) Lyn [24]. Furthermore, the signaling complex has been shown to promote OPC survival [25]. Integrins signal through the same complex to promote OPC proliferation in isolated OPC cultures [26]–[28].

PDGFαR is a phenotypic marker of OPCs [29] and retinal astrocytes [30]. Its ligand, PDGF, is produced by neurons [31] and astrocytes as PDGF-AA [32] and -AB dimers [33]. PDGF stimulates OPC proliferation *in vivo* [34], [35] and *in vitro* [36]–[38] and promotes OPC survival *in vivo* [39] and *in vitro* [25], [40]. PDGF does not mediate survival in cells of the OL-lineage that are more mature than OPCs. The basic fibroblast growth factor (FGF-2) up-regulates PDGFαR expression on early progenitors and, in combination with PDGF, supports long-term proliferation and migration *in vivo* and *in vitro* [41]–[44]. FGF-2 is mitogenic for late progenitors and reversibly blocks terminal differentiation [42], [45]–[48]. FGF-2 is produced by neuronal cells [49], astrocytes [50], [51] and microglia [52]–[54]. Its receptor, FGFR2, is present in cells of the OL lineage [55], [56], neurons, amoeboid microglia [57] and, to a lower extent, in astrocytes [57].

In this study, we tested the hypothesis that PDGF is required for rHIgM22-mediated inhibition of apoptotic signaling and differentiation in isolated OPC cultures. rHIgM22 was identified by its strong binding to mature myelin and rHIgM22 binds much more effectively to mature OLs than to undifferentiated ones. However, the Paz Soldan study suggests that OPCs respond to rHIgM22 more robustly than mature OLs. The ability of either O4 [23] or rHIgM22 [24] to block OPC differentiation also indicates that OPCs express enough rHIgM22 antigen to respond to the IgM.

The *in vivo* reparative action of remyelination promoting IgMs is likely dictated by the immediate microenvironment of the lesion in question. Binding of rHIgM22 to the OL membrane in the presence of PDGF may stimulate OPC proliferation and differentiation and/or promote survival of OPCs and mature oligodendrocytes.

## Materials and Methods

### Chemicals

Human plasma fibronectin (354008) was purchased from BD Biosciences Discovery Labware (Bedford, MA, USA). DMEM (10-017-CV), DMEM/F12 50∶50 (10-090-CV), HBSS (21-022-CV), 0.25% Trypsin (25-050-CV) and sodium pyruvate (25-000-Cl) were from Mediatech (Manassas, VA, USA); penicillin/streptomycin (15140) and N2-supplement (17502-048) were from Invitrogen (Carlsbad, CA, USA); fetal bovine serum (SH30070.03) was from Hyclone (Waltham, MA, USA); sterile water (2F7113) was from Baxter (Deerfield, IL, USA); bovine serum albumin fraction V (A-3294), poly-D-lysine hydrochloride (average mol wt 30,000–70,000) (P7280), sodium periodate (S1878), Fumonisin B1 (F1147), 3,3′,5-Triiodo-L-Thyronine sodium salt (T5515) and D-(+) glucose (G5767) were from Sigma (St. Louis, MO, USA); FGF-2 (01–106) and PDGF-AA (01–309) were from Millipore (Temecula, CA, USA). Ethanol (E200, 111000200) was purchased from Pharmco-Aaper (Brookfield, CT, USA).

### Animals

Pregnant Sprague Dawley rats were purchased from Harlan Laboratories (Madison, WI, USA) and housed in Mayo Clinic’s animal care facility.

Animal protocols were approved by the Mayo Clinic Institutional Animal Care and Use Committee (appointed by the Institutional Official’s delegate, the Board of Governors) and Department of Comparative Medicine provide institutional assurance of compliance with the Animal Welfare Act (Public Law 89–544 and amendments) (protocol number: A29509).

### Cell Culture

#### Mixed glial cultures

We prepared primary mixed glial cultures according to *Asakura et al*. [13]. In brief, we removed brains from newborn P0 to P1 Holtzman Sprague Dawley rats, detached cerebral hemispheres, removed meninges, minced hemispheres and transferred them to Ca ^2+^ - and Mg ^2+^ -free HBSS-containing 5 g/L D-glucose, 3 g/L BSA fraction V, 20 mM HEPES, penicillin/streptomycin (modified HBSS) and 0.05% trypsin. The tissue was incubated in trypsin solution for 30 min at 37°C in a rotary-shaking incubator at 130 rpm. To the digested tissue we added fetal bovine serum (10% final concentration), magnesium sulfate (MgSO4) (0.0164% final concentration) and DNase I (20 µg/ml final concentration) and then incubated it for 10 minutes on ice, gently swirling it after every second minute. The tissue was centrifugated in the cold at 8°C for 5 minutes (200×g) and gently resuspended in modified HBSS (see above). The tissue was further dissociated by trituration through a sterile, 10 ml pipette. The cell suspension was washed by centrifugation (8°C, 10 minutes, 200×g) and plated on poly-D-lysine-coated 75 cm^2^ flasks or 60 mm cell-culture dishes (25 µg/ml poly-D-lysine for 1 h at 37°C and subsequently washed twice with water). Mixed glial cells were plated in DMEM supplemented with 10% fetal bovine serum, 100 U/ml penicillin, 100 µg/ml streptomycin, and 1 mM sodium pyruvate at a density of 8 million cells per 75 cm^2^ flask or 2 million cells per 60 mm dish. Mixed glial cultures were confluent after 2 to 3 days in culture. Resulting mixed glial cultures consisted of 58% astrocytes with 39% OPCs and 3% microglial cells.

#### Isolated OPCs

Mixed glial cultures were maintained for 8 to 12 days in DMEM supplemented with 10% fetal bovine serum, 100 U/ml penicillin, 100 µg/ml streptomycin, and 1 mM sodium pyruvate. Semi-purified OLs were prepared by shaking, using a version of the original protocol from *McCarthy and de Vellis* [58]. Cells were shaken initially for 1 h at 140 rpm to remove microglial, re-fed, and shaken again for 20 h at 37°C at 200 rpm. Microglial and astrocytic contaminants were removed by plating the supernatant cell mixture twice onto Petri dishes for 30 minutes. The cell suspension was centrifuged for 8 minutes at 850 rpm at 10°C, resuspended and cultured for 1 to 7 days in DMEM:F12 (50∶50) culture medium containing 0.1% BSA, 10 ng/ml biotin, 10 ng/ml PDGF-AA, 10 ng/ml FGF-2, 1×N2 supplement, 100 U/ml penicillin, and 100 µg/ml streptomycin (proliferation medium). Acid-washed glass coverslips or 60 mm cell-culture dishes were pre-coated with 25 µg/ml poly-D-lysine for 3 h at 37°C and washed twice with water. Alternatively, glass coverslips or 60 mm dishes were treated with 50 µg/ml human serum fibronectin (BD Biosciences) in sterile HBSS at 37°C for 3 h and rinsed once with water before use. Proliferation medium was changed every second day. After 7 days, culture dishes contained highly enriched populations of OLs (>90%) with 8–10% GFAP-positive astrocytes and 1% CD11b-positive (Ox42) microglia.

### Proliferation Assays

#### A. Tritiated Thymidine incorporation

Rat cerebra were dissociated for primary mixed glial cell culture and plated at 8×10^4^ cells per well in 96 well poly-D-lysine coated culture plates. Cells were maintained in DMEM/10% FBS for 5 days. On day 5 cultures were washed twice with DMEM and switched to modified N2 medium [59]. Serial dilutions of IgM antibodies or PDGF-AA and FGF-2 and 0.75 µCi (1 mCi/ml) of tritium-labeled thymidine (GE Healthcare, Piscataway, NJ) were added to triplicate wells at 24-hour intervals. Cells were washed with DMEM and transferred to Packard Unifiliter-96 GF/C plates using a Packard FilterMate Harvester. Filter-bound tritium was measured using a Packard TopCount Microplate scintillation and luminescence counter (Packard Instrument Co, Meriden, CT). Tritium-uptake studies were verified with 3 independent cell preparations. Data points are mean and standard error of triplicate wells and represent one of three independent tritium-uptake assays using 3 different cultures. We analyzed statistical comparisons of tritium-uptake at all IgM and growth factor concentrations by ANOVA.

#### B. BRDU-incorporation assay

Mixed glial cells were maintained in DMEM/10% FBS for 4 days. On day 4 cultures were washed twice with DMEM and switched to modified N2 medium [59]. IgMs (10 µg/ml each) were added for 48 h from day 4–6. Positive control PDGF and FGF-2 (10 ng/ml each) was added for 24 h from day 5–6. BRDU was added to mixed glial cells for 18 h from day 5–6 in a final concentration of 10 µM. Cells were washed extensively, fixed with 4% PFA for 15 minutes, permeabilized with 0.1% Triton X-100 in PBS for 2 minutes at RT and processed for immunocytochemistry.

#### C. Determination of proliferating cell types

We treated mixed glial cells plated on poly-D-lysine (PDL) 7 days after isolation with rHIgM22 or human isotype control IgM (10 µg/ml each) for another 48 h in chemical defined media without PDGF/FGF-2. The percentage of proliferating astrocytes, microglia/macrophages, and oligodendrocytes in mixed glial cultures were determined by analyzing the extent of co-localization between proliferation marker Ki-67 and cell-specific markers GFAP (astrocytes), CD68 (microglia, macrophages) and Olig-1 and Olig-2 (oligodendrocytes) in immunocytochemistry. We repeated experiments with mixed glial cultures from three independent glial preparations.

#### D. Analysis of expression levels after rHIgM22-treatment

We treated mixed glial cultures identical to experiments described as under B. Levels of expression of astrocytic, microglial and oligodendroglial markers were analyzed via Western blots. PDGF/FGF-2 was used as a positive control for oligodendrocyte progenitor proliferation. We repeated experiments with mixed glial cultures from three independent glial preparations.

#### E. Chemical inhibitors used for inhibition of rHIgM22-mediated stimulation of expression of OPC markers

Similar to experiments described above, mixed glial cells on PDL were treated 7 days after isolation for another 48 h with rHIgM22 plus chemical inhibitors PDGFR kinase inhibitor AG1296 (20 µM), SFK inhibitor PP2 (10 µM), MEK kinase inhibitor UO126 (10 µM) or vehicle (DMSO). We analyzed different treatment groups via Western blots. Experiments were repeated with mixed glial cultures from three independent glial preparations.

### Analysis of rHIgM22-mediated Signaling in OPCs, Modulation of OPC Cell Death and OPC Differentiation

OPC cultures grown in the presence or absence of PDGF/FGF-2 (10 ng/ml PDGF; 10 ng/ml FGF-2) were characterized via immunocytochemistry at day 7 with OPC/OL markers A2B5, O4 and MBP.

Isolated OPC cultures were treated with rHIgM22 or human isotype control IgM (10 µg/ml each) in the presence of absence of growth factors PDGF and FGF-2 (10 ng/ml PDGF; 10 ng/ml FGF-2) for 1 to 7 days on fibronectin. Human isotype control IgM or rHIgM22 were present during the whole experiment and consistently added to both treatment groups upon media exchange (every second day). We analyzed treatment groups via Western blots. Experiments were repeated with cells of the OL-lineage from three independent glial preparations.

### Fluorescence Microscopy Studies

For epifluorescence microscopy we used an Olympus IX70 microscope equipped with a PE 94 cold stage (Linkam Scientific Instruments, Tadworth, Surrey, UK), a QuantEM 512SC CCD camera and a 60× 1.4 NA lens. Quantitation of images was performed using the MetaMorph image-processing program (Molecular Devices, Sunnyvale, CA) as described [60]. All photomicrographs were exposed and processed identically for a given fluorophore. Primary and secondary antibodies were used for immunocytochemistry (IC) in concentrations as listed (Table1, Table 2). To avoid spill-over in double-labeling experiments we used the combination of AF488-labeled (green) and AF647-labeled (far-red) secondary antibodies.

**Table 1:** Primary antibodies.

Antibody	Host, monoclonal or polyclonal, clone	Concentration/dilution	Company and Catalog number
A2B5, OPC	Mouse, monoclonal	10 µg/ml (IC)	In house
O4, OPC, immature and mature OL	Mouse, monoclonal	10 µg/ml (IC)	In house
rHIgM22	Human, monoclonal	10 µg/ml (IC)	In house
ChromPure human IgM, human control IgM	Human, oligoclonal	10 µg/ml (IC)	Jackson ImmunoResearch, #009-000-012
MBP, mature OL	Rabbit, polyclonal	1∶5000 (WB)1∶100 (IC)	Millipore, AB980
phospho-p44/42 MAP Kinase antibody (Thr202/Tyr204), MAP kinase pathway	Rabbit, polyclonal	1∶1000 (WB)	Cell signaling, #9101
p44/42 MAP Kinase antibody (ERK1/2), MAP kinase pathway	Rabbit, polyclonal	1∶1000 (WB)	Cell signaling, #9102
pSrc(pY416), phospho Src family kinases	Rabbit, polyclonal	1∶1000 (WB)	Cell signaling, #2101
c-Src, Src family kinase	Rabbit, polyclonal	1∶1000 (WB)	Cell signaling, #2108
Lyn, Src family kinase	Mouse, monoclonal, clone H6	1∶200 (WB)	Santa cruz, sc-7274
Fyn, Src family kinase	Mouse, monoclonal, clone 1S	1∶1000 (WB)	Millipore, MAB8900
PDGFαR, OPC	Rabbit, polyclonal	1∶200 (WB)	Santa cruz, sc-338
CNPase, early OL differentiation marker	Mouse, monoclonal, clone 11-5B	1∶1000 (WB)	Sigma, C5922
NG2, OPC	Rabbit, polyclonal	1∶1000 (WB)	Millipore, AB5320
Olig-1, OPC	Rabbit, polyclonal	1∶1000 (WB)1∶100 (IC)	Chemicon, AB15620
Olig-2, OPC and immature OL	Rabbit, polyclonal	1∶1000 (WB)1∶100 (IC)	Chemicon, AB9610
Ki-67, proliferation	Mouse, monoclonal, clone B56	1∶1000 (WB), 1∶100 (IC)	BD#550609
Ki-67, proliferation	Rabbit, polyclonal	1∶50 (IC)	Abcam, Ab16667
Cleaved caspase-3, apoptosis	Rabbit, polyclonal	1∶1000 (WB)	Cell signaling, #9661
Cleaved caspase-9, apoptosis	Rabbit, polyclonal	1∶1000 (WB)	Cell signaling, #9507
GFAP, Astrocytes	Mouse, monoclonal, clone 52/GFAPl	1∶1000 (WB)	BD#610565
GFAP, Astrocytes	Rabbit, polyclonal	1∶1000 (IC)	Abcam, Ab7779
CD68, Monocyte/Macrophage	Mouse, monoclonal,Clone ED1	1∶1000 (WB)1∶100 (IC)	Millipore, MAB1435
β-actin, cytoskeleton	Rabbit, polyclonal	1∶1000 (WB)	Cell signaling, #4967

**Table 2:** Secondary antibodies.

Antibody	Concentration/dilution	Company and Catalog number
Goat anti rabbit IgG (H+L), horseradish peroxidase(HRP) conjugated	1∶5000 (WB)	Millipore, AP132P
Goat anti mouse IgG (H+L), horseradish peroxidase(HRP) conjugated	1∶5000 (WB)	Millipore, AP124P
Goat anti mouse IgM (H+L), Alexa Fluor 488 conjugated	1∶250 (IC)	Invitrogen, A11029
Goat anti rabbit IgG (H+L), Alexa Fluor 488 conjugated	1∶250 (IC)	Invitrogen, A11034
Goat anti rabbit IgG (H+L), Alexa Fluor 647 conjugated	1∶250 (IC)	Invitrogen, A21245
Goat anti mouse IgG (H+L), Alexa Fluor 488 conjugated	1∶250 (IC)	Invitrogen, A21121
Goat anti mouse IgG (H+L), Alexa Fluor 647 conjugated	1∶250 (IC)	Invitrogen, A21235

### Western Blotting

Isolated OLs were incubated for 1 to 7 days with 5 µg/ml rHIgM22 or 5 µg/ml human isotype control IgM in 60 mm cell-culture dishes on fibronectin. Fresh IgM antibodies were added every second day and triplicates used for each condition. Cells were washed three times with ice-cold Ca ^2+^ - and Mg ^2+^ -free HBSS and lysed on ice with RIPA buffer supplemented with 1 mM Na_3_VO_4_, 10 mM NaF, and a protease-inhibitor mixture. Cells lysates were transferred to microfuge tubes and incubated on ice. Lysates were homogenized by trituration through a 21-gauge needle and centrifuged at 14,000 rpm for 5 minutes at 4°C to separate from detergent-insoluble material. For Western blotting, 10–20 µg protein per lane were analyzed as described [13]. For quantitation, 3 to 5 experiments each from independent OL cultures were used with subsequent densitometric analysis of Western blots.

### Statistical Analyses

All experiments were conducted in triplicate and carried out on at least two occasions. Sigma Plot and Sigma Stat were used for statistical analysis. We used Student’s t-test distribution or ANOVA analysis to determine levels of significance for comparison between all cell lines. A value of 0.05 was considered significant. All analyses were performed in a “blinded” fashion without knowledge of the treatment group.

## Results

### rHIgM22 Drives Oligodendrocyte Progenitor Proliferation in Mixed Glial, but not in Isolated OPC

**Figure 1:**
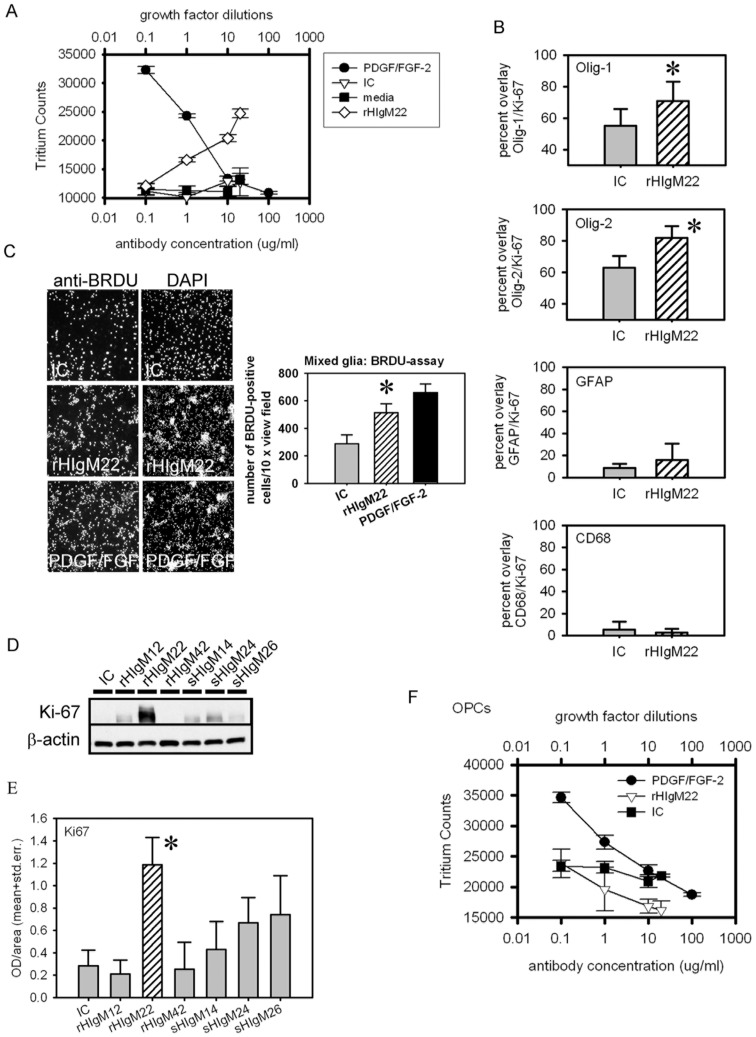
rHIgM22 Induced Thymidine Uptake in Primary Mixed Glial Cultures, but not in isolated OPCs. **A-C Mixed glial cultures.** Mixed glial cells were maintained in serum-containing medium for 5 days prior to the addition of IgMs and/or growth factors PDGF and FGF-2 in serum free media for 48 h. **A**. Tritiated thymidine, IgMs or PDGF/FGF-2 were added at day 5 in a serum-free medium. The mitogens PDGFAA and FGF2 were included as positive controls and at the highest concentrations consistently increased tritium uptake 3-fold over medium alone. PDGFAA (20 ng/ml) and FGF2 (10 ng/ml) were added at serial 10-fold dilutions. rHIgM22 induced thymidine uptake by progenitor oligodendrocytes at both 20 µg/ml, 10 µg/ml and 1 µg/ml over medium alone or human isotype control IgM (p<0.001 by one way ANOVA). In contrast isotype control IgM, which does not promote remyelination *in vivo*, did not induce uptake of thymidine. Data points are mean and standard error of triplicate wells and represent one of three independent tritium uptake assays using 3 different cultures. **B.** Quantitative analysis of immunocytochemical data from mixed glial cultures treated with human isotype control IgM (IC) or rHIgM22 showing the extent of co-localization between glial markers Olig-1, Olig-2, GFAP, CD68 and proliferation marker Ki-67 in percent of cells positive for glial marker (e.g. Olig-1). **C.** BRDU-assay in mixed glial cultures after treatment with IgMs for 48 h (10 µg/ml each) or 24 h of PDGF/FGF-2. Representative images show the extent of BRDU-positive cells (anti-BRDU) relative to total cell numbers (DAPI) in isotype control (IC), rHIgM22 and PDGF/FGF-2 treated mixed glia plus quantitation of BRDU-positive cells per 15×field for all treatment groups. **D+E**. Mixed glial cultures were treated with rHIgM22 or control IgMs for 7 days in serum-free medium. D. Representative Western blots from one of three independent experiments show levels of proliferation marker Ki-67 in addition to loading control beta-actin. E. Quantitative analysis of Western blots from experiments shown in D. Data are presented as mean ± S.D. (*n = *3). * *p*<0.05 compared to controls. **F.** Tritiated thymidine, human isotype control (IC), rHIgM22 or PDGF/FGF-2 were added at day 5 identical to under A. At the highest concentrations PDGF-AA and FGF-2 consistently increased tritium uptake 2-fold over both IgM treatments. rHIgM22 did not induce thymidine uptake by progenitor oligodendrocytes at all concentrations used compared to human isotype control IgM. Data points are mean and standard error of triplicate wells and represent one of three independent tritium uptake assays using 3 different cultures.

rHIgM22 significantly enhanced tritium-labeled thymidine uptake in mixed glial cultures at concentrations of 1, 10 and 20 µg/ml compared to a human isotype control IgM or media alone (Table S1; Fig. 1A). We observed no significant difference between human isotype control IgM and media at any concentration. The presence of PDGF and FGF-2 in the media significantly enhanced tritiated-thymidine uptake (20 ng/ml PDGF, 10 ng/ml FGF-2, and 200 ng/ml PDGF, 100 ng/ml FGF-2) compared to human isotype control IgM or medium (Fig. 1A, Table S1).

To identify the cell types in mixed glial cultures stimulated to proliferation by rHIgM22, we analyzed the extent of co-localization between the proliferation marker Ki-67 and cell type specific markers. At 48 h post-treatment with rHIgM22 or human isotype control IgM (IC) we used immunocytochemistry to identify GFAP-positive astrocytes, CD68-positive microglia/macrophages, and Olig-1- and Olig-2-positive oligodendrocytes. rHIgM22 significantly enhanced co-localization between the oligodendrocyte markers Olig-2 and Olig-1 with Ki-67 (Olig-2, student’s t-test, p<0.005; Olig-1, student’s t-test, p<0.05) compared to human isotype control IgM (Fig. 1B, Fig. S1, Table 3). We observed no significant change in co-localization between GFAP and Ki-67 or between CD68 and Ki-67 after rHIgM22 treatment compared to controls (Fig.1B; Fig. S1, Table 3). The expression levels of astrocytic, microglial and OPC markers in mixed glial cultures were also determined by Western blot after treatment with rHIgM22 or controls.

**Table 3:** Proliferating cell types upon IgM-treatment.

	Isotype control treatment			
	Percent of Olig-1-positive cells (± std.-dev.)	Percent of Olig-2-positive cells (± std.-dev.)	Percent of GFAP-positive cells (± std.-dev.)	Percent of CD68-positive cells (± std.-dev.)
Double-labeled with Ki-67	55±11	63±7	8±4	5±7
	**rHIgM22 treatment**			
	**Percent of Olig-1-positive cells (± std.-dev.)**	**Percent of Olig-2-positive cells (± std.-dev.)**	**Percent of GFAP-positive cells (± std.-dev.)**	**Percent of CD68-positive cells (± std.-dev.)**
Double-labeled with Ki-67	71±12	82±8	16±14	3±3

We detected significantly increased expression levels of Olig-1 and Olig-2 in rHIgM22-treated groups (student’s t-test, p<0.005 for Olig-1; p<0.01 for Olig-2). The addition of PDGF/FGF-2 (10 ng/ml each) increased the expression levels of both OPC markers over rHIgM22-treatment, but this was not statistically significant. rHIgM22 slightly increased the expression of GFAP and CD68 in mixed glia, which was also not statistically significant (Fig. S2 A, B). Despite slightly increased expression levels of GFAP and CD68, rHIgM22 did not stimulate proliferation of astrocytes or microglia. The increase may be explained by astrocytes and microglia being in a reactive state.

To further support the stimulatory capacity of rHIgM22 to promote OPC proliferation, we used a BRDU-incorporation assay as a third independent method. Treatment of mixed glia with rHIgM22 resulted in a 1.8-fold increase in BRDU-positive cells relative to a human isotype control (Fig. 1 C). PDGF and FGF-2 (10 ng/ml each) increased the extent of BRDU-positive cells 2.2 fold compared to a human isotype control (Fig. 1 C). Both changes were highly significant (student’s t-test, p<2.3900e-8 for rHIgM22 vs isotype control; p = 0.0000 for PDGF/FGF-2 vs isotype control). Nuclear staining with DAPI demonstrated substantially higher total numbers of cells after rHIgM22treatment compared to isotype control-treated glial cells (Fig. 1 C), which further confirms our data.

In addition, we measured expression levels of Ki-67 in mixed glial cultures after 7 days of treatment with rHIgM22 compared to human IgM controls (Fig. 1 D, E). rHIgM22 substantially increased levels of Ki-67 compared to all tested human control IgMs. This was statistically significant compared to a recombinant human control IgM, rHIgM12 (student’s t-test, p = 0.02), and the polyclonal human isotype control (IC) (student’s t-test, p = 0.03).

Because these first studies indicated that OPCs were the mixed glial cell type responsive to rHIgM22, we repeated tritiated-thymidine uptake and OPC specific expression studies in isolated OPCs. Within a concentration range of 1–20 µg/ml, rHIgM22 reduced, rather than stimulated, tritiated-thymidine uptake in isolated OPCs compared to a human isotype control (Fig. 1 F). Analysis (one-way Anova) revealed statistical significance between rHIgM22 and human isotype control-treated OPCs at the highest antibody concentration used (20 µg/ml) (rHIgM22 vs IC, one-way Anova, p = 0.02). Tritiated thymidine uptake at lower rHIgM22 concentrations compared to isotype control-treated OPCs were not statistically different. This suggests that rHIgM22 has an inhibitory potential on OPC proliferation in monoculture when used at antibody concentrations >10 µg/ml in the absence of externally added PDGF and FGF-2.

The addition of PDGF/FGF significantly stimulated tritiated-thymidine uptake into OPCs, demonstrating the intact proliferative capacity and viability of cells used in the assay (Fig. 1 F). The presence of rHIgM22 in OPC cultures for 1 to 7 days did not alter the expression levels of PDGFαR, NG2 and Olig-2. OPCs were maintained in a defined media without PDGF or FGF-2 (Fig. S2 C).

These results suggest that rHIgM22 does not induce OPC proliferation directly but perhaps via an indirect mechanism in mixed glial culture involving astrocytes and/or microglia. Soluble factors secreted by astrocytes and/or microglia or direct cell contact may be necessary co-factors for rHIgM22-induced OPC proliferation.

### The PDGF-pathway is Required for rHIgM22 Stimulation of OPC Proliferation

PDGF is a well-characterized and potent mitogen that stimulates proliferation of early OPCs [34]–[38]. FGF-2 promotes proliferation of A2B5-positive and O4-positive OPCs independently of PDGF in isolated neonatal cultures [61], [62]. In addition, the major mitogen identified in axolemma-enriched fractions accounting for OPC proliferation was acidic FGF (aFGF) followed by FGF-2, but not PDGF [63].

Astrocytes and microglia may provide PDGF in mixed glial cultures to support rHIgM22-induced OPC proliferation. We directly tested whether PDGF is necessary for rHIgM22-mediated promotion of OPC proliferation by blocking the PDGF pathway with the chemical inhibitor AG1296, which blocks PDGFR kinase. Use of AG1296 reduces OPC proliferation in rHIgM22 and isotype control-treated mixed glial cells. However, if rHIgM22 promotes OPC proliferation independently from the PDGF-pathway (e.g. via FGFRs), then a significant difference in the extent of proliferating OPCs should be expected in AG1296-treated cells combined with rHIgM22 compared to AG1296 plus isotype control IgM.

**Figure 2:**
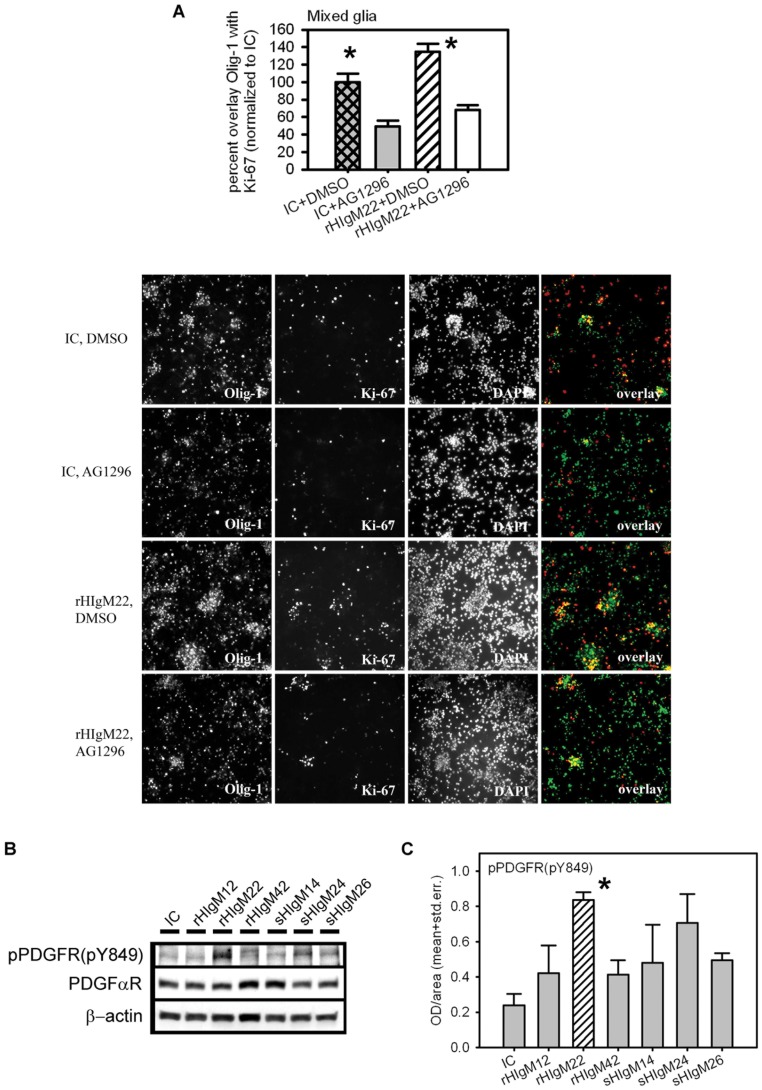
PDGF-mediated signal transduction necessary for rHIgM22-mediated promotion of OPC proliferation. Mixed glial cells were maintained in serum-containing medium for 5 days after tissue isolation prior to the addition of rHIgM22 plus PDGFR kinase inhibitor AG1296 (10 µM) or vehicle (DMSO) for 48 h in serum-free media. **A.** Double immunofluorescence with oligodendrocyte marker Olig-1 and proliferation marker Ki-67 in mixed glial cultures treated with IgMs rHIgM22 or isotype control (IC) (10 µg/ml each) in the presence of AG1296 or DMSO. Bar graphs show the extent of double-labeling between Olig-1 and Ki-67 in each treatment group relative to isotype control treatment (100%). **B.** Representative Western blots from one of three independent experiments in mixed glia show levels of activated PDGF receptor (pTyr^849^), PDGFαR and beta-actin as loading control after 7 days of treatment with rHIgM22 or human control IgMs in serum-free media. **C.** Quantitative analysis from 3 independent experiments under the conditions described above. Background is subtracted from each value and normalized against β-actin. Data are presented as mean ± S.D. (*n = *3). * *p*<0.05 compared to controls.

In mixed glial cultures treated with PDGFR-kinase inhibitor, rHIgM22 induced 50% fewer Ki-67/Olig-1-positive OPCs compared to inhibitor vehicle DMSO (Fig. 2A). The percent of Ki-67/Olig-1-positive OPCs induced by rHIgM22 or human control IgM in the presence of PDGFR-kinase inhibitor did not differ. However, rHIgM22 with the inhibitor vehicle stimulated proliferation of OPCs by 34% over the control IgM and inhibitor vehicle.

To further support the involvement of PDGFαR in rHIgM22-stimulated OPC proliferation, we assessed levels of phosphorylated PDGFαR at tyrosine 849 (pY849) in IgM-treated mixed glial cultures. In mixed glial cultures, rHIgM22 increased levels of phosphorylated PDGFαR (pY849) 1.7–6.5 fold after normalization to total PDGFαR compared to all tested human IgMs after 7 days of treatment (Fig. 2 B, C). This was significant when compared to human isotype control (student’s t-test, p = 0.002), to a recombinant human control IgM, rHIgM42 (student’s t-test, p = 0.01) and a serum-derived human control IgM, sHIgM26 (student’s t-test, p = 0.005). Autophosphorylation of PDGFαR at Tyr^849^ or the corresponding Tyr^857^ in PDGFβR regulates the catalytic activity of the kinase [64]. Mutation of this tyrosine to a phenylalanine residue gives a receptor with a lowered kinase activity [65], [66], suggesting that phosphorylation of Tyr^849^/Tyr^857^ in PDGFαR respective PDGFβR is important for activation of the kinase [64].

However, in isolated OPC monoculture, the addition of PDGF and FGF-2 combined with rHIgM22 did not stimulate proliferation over controls (PDGF/FGF-2 combined with IC; PDGF/FGF-2 combined with medium) as assessed by BRDU-incorporation (Fig. S5). This indicates that PDGF is necessary, but not sufficient, for IgM-stimulated OPC proliferation. Other soluble astrocytic or microglial factors or direct contact of OPCs to astrocytes may be required in addition to PDGF to achieve the stimulatory effect of rHIgM22 on OPC proliferation.

### rHIgM22-mediated Induction and Activation of Signaling Molecules Lyn, ERK1 and ERK2 on OPCs Require PDGF and FGF-2

To further investigate the role of PDGF combined with FGF-2 on rHIgM22-stimulated OPC proliferation, we studied isolated OPC cultures in which the levels of PDGF and FGF-2 could be tightly controlled. PDGF used in combination with FGF-2 kept OPCs in an undifferentiated, proliferative state for the detection of long-term effects days after IgM administration. FGF-2 reversibly blocks terminal OPC differentiation and up-regulates PDGFαR expression on early (A2B5+) progenitors [41]–[48].

**Figure 3:**
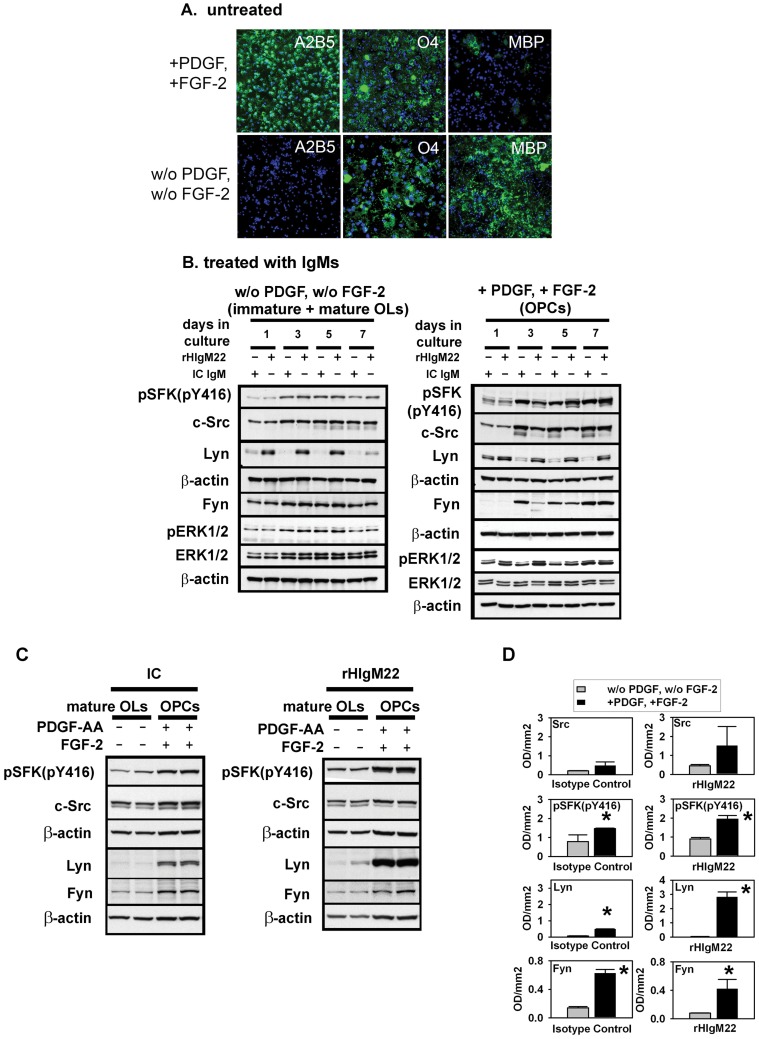
rHIgM22-mediated signal transduction in OPCs requires PDGF and FGF-2. A. OLs were cultured for 7 days on fibronectin in the presence or absence of growth factors PDGF-AA and FGF-2. OLs were pre-labeled with rHIgM22 (10 µg/ml), O4 (10 µg/ml) or A2B5 (10 µg/ml) for 30 minutes in HMEM at 8 °C, fixed and processed for immunocytochemistry including DAPI-staining. For MBP-staining, OLs were fixed, permeabilized and stained with anti-MBP (MBP staining = green, DAPI = blue). Results were repeated 3 times with identical results. **B+C.** OLs were cultured identical to A and treated with rHIgM22 (5 µg/ml) or isotype-control IgM (IC) (5 µg/ml) in the absence or presence of growth factors PDGF-AA and FGF-2. **B.** Comparison of OPCs with OPCs (+PDGF/FGF-2) and mature OLs with mature OLs (w/o PDGF/w/o FGF-2). Representative blots showing the levels of phospho-SFKs (pY416), phospho-p44/42 MAP Kinase (pT202/pY204) (pERK1/2), Lyn, Fyn, c-Src, p44/42 MAP Kinase (ERK1/2) and β-actin as a loading control at day 1, 3, 5 and 7 in culture. **C.** Comparison of OPCs and mature OLs on the same Western blot. Representative Western blots showing levels of activated SFKs (pY416), Lyn, Fyn, c-Src and β-actin as a loading control at day 7 in culture. Each sample is loaded as a doublet. **D.** Quantitative analysis of 3 independent experiments as described in C. Background is subtracted from each value and normalized against β-actin levels. Data are presented as mean ± S.D. (*n = *3). * *p*<0.05 compared to controls.

After 7 days in culture, we used immunocytochemistry to characterize isolated OPC/OL cultures grown with and without PDGF/FGF-2. OPC cultures treated with PDGF/FGF-2 contained more than 90% of OL-lineage cells still at the progenitor stage and positive for early OPC marker A2B5 and late OPC marker/immature OL marker O4. The cultures contained less than 5% mature MBP-positive OLs. In contrast, cultures maintained without PDGF and FGF-2 contained more than 90% MBP-positive OLs and less than 5% A2B5-positive OPCs (Fig. 3A).

In OPC cultures maintained with PDGF and FGF, the addition of rHIgM22 activated MAP (Mitogen-activated protein) kinases ERK1 and ERK2 at day 1 and day 3 in culture (Student’s t-test, p<0.05) and Src family kinases (SFKs) [27], [62], [67] at days 5 and 7 in culture (Student’s t-test, p<0.05). We assessed SFK activation using an antibody detecting a common phosphorylated epitope at tyrosin 416 (pY416) in c-Src, Lyn and Fyn present in OPCs [68]–[70]. The SFK Lyn, a common regulator of proliferation and apoptosis [71]–[75], is involved in PDGF-mediated promotion of OPC proliferation [27]. Treating OPCs with rHIgM22 in the presence of growth factors also induced Lyn expression, which was significantly elevated at days 1, 3 and 7 compared to control (Student’s t-test, p<0.005) (Fig. 3B, Fig. S3+S4) and reduced the expression levels of c-Src and Fyn (Fig. 3B, Fig. S3+S4).

In OLs deprived of PDGF and FGF-2, rHIgM22 induced significant Lyn expression at days 3 and day 5 in culture (Student’s t-test, p<0.05) with expression levels decreasing over time. In contrast to signal transduction observed in OPCs, rHIgM22 did not affect the activation of SFKs, ERK1 and ERK2 or the expression levels of c-Src or Fyn in immature or mature OLs (Fig. 3B, Fig. S3+S4). These data indicate that early (A2B5+/O4−) and late (A2B5+/O4+) progenitors, rather than mature MBP-positive OLs, respond to rHIgM22 treatment.

We directly compared effects of IgM addition to OPCs or mature OLs at days 7 in culture using the same Western blot. Independent of the added IgM, OPCs cultured with PDGF and FGF-2 expressed higher levels of SFKs Lyn and Fyn and contained a higher overall activation level of SFKs (pY416) compared to mature OLs (Fig. 3 C, D). Importantly, rHIgM22 combined with both growth factors potentiated the induction of Lyn expression in OPCs compared to human isotype control IgM suggesting a synergistic effect of rHIgM22 and PDGF/FGF-2 on the up regulation of Lyn expression in OPCs (Fig. 3 C, D).

These results highlight the importance of PDGF/FGF-2 for rHIgM22-mediated signal transduction in OPCs. The growth factors maintain OPCs in an immature proliferative stage that responds to rHIgM22 and appears to synergize with rHIgM22 to enhance Lyn expression. The data further suggest that PDGF and FGF-2 modulate rHIgM22-mediated changes in OL differentiation and survival.

### PDGF and FGF-2 are Essential for rHIgM22-mediated Inhibition of OPC Apoptotic Signaling and Differentiation

The ability of rHIgM22 to mediate changes in SFK activation and expression levels suggests a possible role for rHIgM22 in OPC survival and differentiation. To address this, we analyzed the effect of rHIgM22 on OPC/OL caspase and OL antigen expression by Western blot.

**Figure 4:**
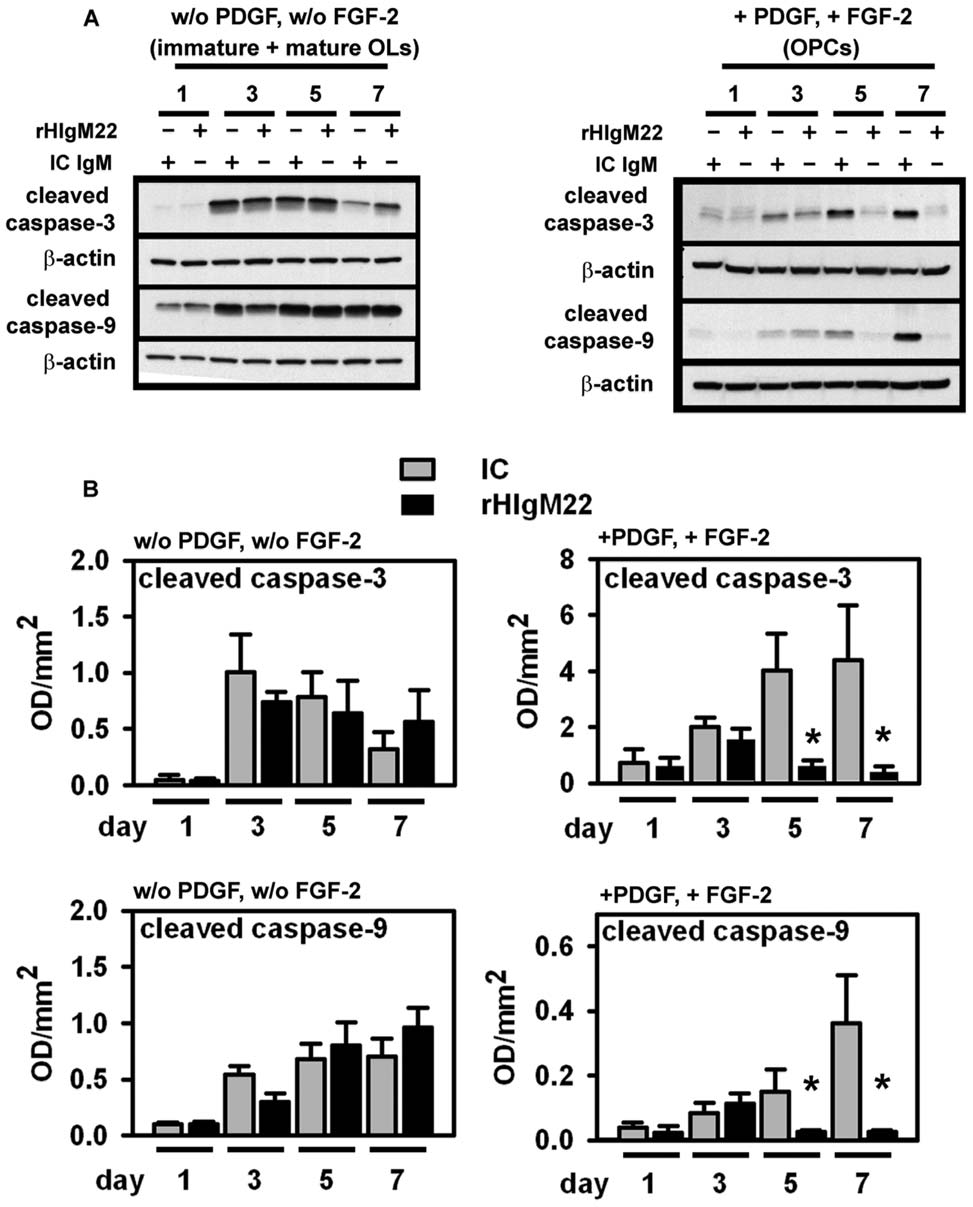
Synergistic inhibition of apoptotic signaling in OPCs by rHIgM22 and PDGF/FGF-2. A+B: OLs were cultured for 7 days on fibronectin with rHIgM22 (5 µg/ml) or isotype-control IgM (IC) (5 µg/ml) in the absence or presence of growth factors PDGF-AA and FGF-2. **A:** Representative Western blots of cleaved caspase-3 (19 kDa fragment), cleaved caspase-9 (17 kDa fragment) and β-actin from OLs cultured for 1–7 days on fibronectin with rHIgM22 (5 µg/ml) or isotype-control HIgM (IC IgM) (5 µg/ml) in the absence or presence of growth factors PDGF-AA and FGF-2. **B:** Quantitative analysis from 3 independent experiments under the conditions described above. Background is subtracted from each value and normalized against β-actin. Data are presented as mean ± S.D. (*n = *3). *p<0.05 compared to controls.

**Figure 5:**
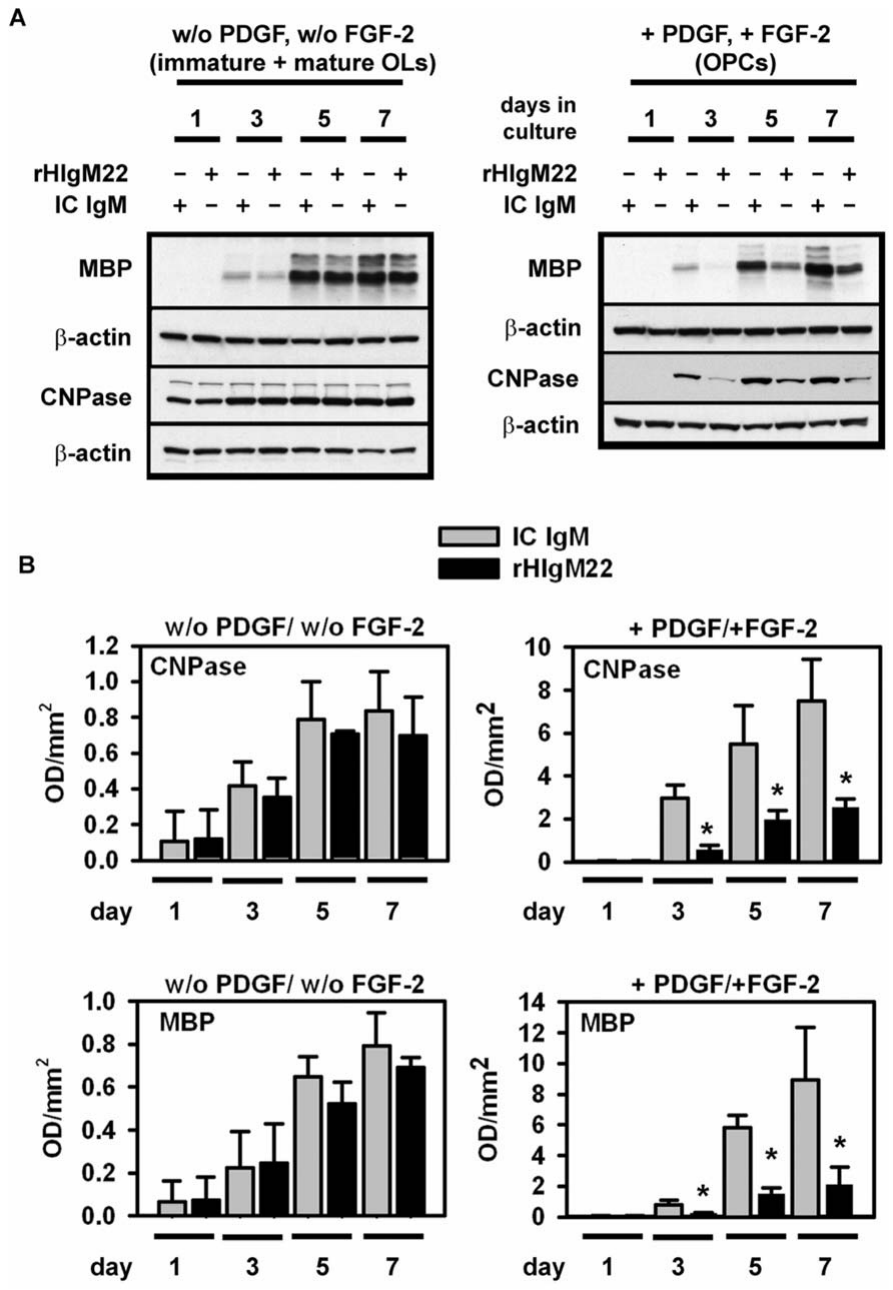
RHIgM22 reduces expression of OL differentiation markers in the presence of PDGF/FGF-2. A: OLs were cultured for 1–7 days on fibronectin with rHIgM22 (5 µg/ml) or the human isotype control IgM (IC) (5 µg/ml) in the absence or presence of growth factors PDGF-AA and FGF-2. Representative Western blots showing the levels of CNPase, MBP and β-actin as a loading control. **B:** Quantitative analysis of 3 independent experiments as described in A. Background is subtracted from each value and normalized against β-actin levels. Data are presented as mean ± S.D. (*n = *3). * *p*<0.05 compared to controls. IC: isotype control IgM.

In OL cultures grown in the absence of PDGF and FGF-2, the highest levels of cleaved caspase-3 were measured between days 3 and 5 in culture, whereas the cleaved caspase-9 level continued to increase through day 7 in culture. The addition of rHIgM22 or human isotype control IgM did not affect the expression profiles of cleaved caspase-3 or caspase-9 in immature and mature OLs (Fig. 4 A, B). In addition, rHIgM22 did not change the expression of OL-differentiation markers MBP and CNPase in cells grown without PDGF and FGF-2 (Fig. 5 A, B). We noted a high percentage of apoptotic OLs during differentiation in the absence of PDGF and FGF-2.

In stark contrast, the addition of rHIgM22 to OPCs maintained in the presence of PDGF and FGF-2 significantly reduced cleaved caspase-3 and cleaved caspase-9 levels compared to human isotype control IgM. This was most pronounced at days 5 and day 7 in culture, when cells were still at the late progenitor stage (A2B5+, O4+) (Fig. 4 A, B). Similarly, the addition of rHIgM22 to OPCs maintained with PDGF and FGF-2 significantly reduced MBP and CNPase levels between days 3 and 7 in culture. We did not observe this reduction in MBP and CNPase expression in OPCs treated with human isotype control IgM (Fig. 5 A, B).

These results suggest that the interaction of rHIgM22 with OPCs but not mature OLs delays differentiation and prolongs proliferation. The data also show that rHIgM22 protects OPCs from apoptotic signaling, which may represent a synergy with PDGF and FGF.

## Discussion

Polyclonal and monoclonal IgMs that bind to CNS antigens promote remyelination in the TMEV- and lysolecithin-induced animal models of MS [15], [18]. A single low dose of rHIgM22 (25 µg/kg) promotes substantial remyelination of demyelinated lesions [18], [19] suggesting that the effect of the IgM is amplified in some manner by endogenous mechanisms of remyelination. Strong evidence confirms that rHIgM22 crosses the blood brain barrier and targets the demyelinated lesion [76]. Within IgM-remyelinated lesions, immunohistochemical and ultrastructural electron-microscopy studies reveal remyelinated axons, identified by their relatively thin myelin sheaths compared to normal myelin [15], [16], [18], [21]. Prior evidence demonstrated that all remyelination promoting IgMs induce Ca^2+^-influx in OPCs and immature OLs [20]. Even IgMs that bind primarily to mature OLs and myelin only elicit a response from immature OLs and OPCs [20]. Immature proliferative stages of OLs may mediate IgM-stimulated remyelination. A mechanism that works through endogenous OPC proliferation would be consistent with the mechanism of remyelination in most animal models, where adult OPCs, in contrast to surviving mature OLs, are the major source of remyelinating OLs [8], [77]–[81].

We have previously shown that rHIgM22 inhibits apoptotic signaling and differentiation of OLs in culture. In the present study, we have extended these observations to demonstrate that rHIgM22 induces OPC proliferation and that this effect depends upon the growth factor PDGF.

Only mixed glial cultures consisting of astrocytes, OPCs and microglial cells demonstrate observable rHIgM22-mediated OPC proliferation; isolated OPCs do not. This suggests that factors produced by astrocytes or direct contact co-stimulate the proliferative response. rHIgM22 does not bind to astrocytes detected by immunocytochemistry. The fact that an effect is evident in mixed glial but not in isolated cultures supports other studies demonstrating IgM-mediated effects on OPC differentiation in mixed glia only [22].

The fact that most glial cell-secreted PDGF derives from astrocytes [32], [33] further supports a possible role for secreted astrocytic factors in IgM-stimulated OPC proliferation and remyelination. Microglia can be stimulated by Fc receptor binding with IgMs having a more potent effect than IgGs [82]. However, our data favor a potential role of rHIgM22-mediated stimulation of astrocytes, which in turn may promote OPC proliferation. In addition, the low percentage of microglia in our mixed glial cultures (3%) compared to astrocytes (58%) does not support a potential major role of microglia in rHIgM22-mediated OPC proliferation. Remyelination promoting IgMs induce a Ca^2+^-influx into astrocytes and oligodendroglia but not microglia in mixed glial cultures [20]. Nor has a response to rHIgM22 been observed in isolated microglial cultures [24].

The role of PDGF, a mitogen and growth factor secreted by astrocytes and neurons, in OPC proliferation is well characterized [34]–. We have shown that the OL membrane-signaling complex responsible for rHIgM22-mediated responses in OPCs consists of the PDGFαR, integrin αvβ3 and the SFK Lyn [24], which led us to hypothesize that PDGF is necessary for rHIgM22-mediated OPC proliferation. In support of our hypothesis, we showed rHIgM22-mediated activation of the PDGFαR in mixed glial cultures. Blocking the PDGF-signaling pathway prevents rHIgM22-stimulated OPC proliferation in mixed glia. It is noteworthy that PDGF is necessary, but not sufficient, for IgM-stimulated OPC proliferation. Other soluble factors or direct contact of OPCs to astrocytes are required in addition to PDGF to achieve this effect.

Whether IgM-mediated remyelination *in vivo* requires PDGF is unclear. The physiological PDGF concentration in embryonic CNS is below 1 ng/ml [83]. This concentration, at least *in vitro*, is insufficient to induce OPC proliferation [84] and survival [85], [86] and suggests the need for additional signaling cues to generate cellular responses to PDGF *in vivo* [26]. The precise PDGF concentration in a demyelinated lesion is unknown. In murine hepatitis virus demyelinated spinal cord lesions, however, PDGF-A mRNA transcripts are ∼3-fold up regulated over controls [87]. The PDGF-A immunoreactivity appears to increase locally in lesion areas and is primarily associated with reactive GFAP-positive astrocytes but not with neurons [87]. Furthermore, in the cuprizone-induced demyelination model, *hPDGF-A* transgenic mice show elevated OPC density and proliferation in the corpus callosum during acute demyelination and reduced levels of apoptosis during the recovery period after chronic demyelination [88]. Therefore, PDGF may support OPC proliferation and survival and promote remyelination in demyelinated lesions.

The mitogens neurotrophin-3 (NT3), insulin-like growth factors (IGFs), growth-regulated oncogene-α (GRO-α) and FGF-2 can facilitate PDGF-induced proliferation in OPCs [41], [42], [89]–[91]. Likewise, rHIgM22 may enable PDGF by acting directly on OPCs as a stimulating co-factor/modulator of PDGF-mediated proliferation. At lower PDGF concentrations, rHIgM22 may rearrange the OL membrane to create a responsive signaling complex. This is what we initially suggested when we observed that increasing concentrations of rHIgM22 induces tritiated-thymidine uptake in progenitor clusters in mixed glial cultures. Alternatively, rHIgM22 may act indirectly on astrocytes by stimulating production and secretion of growth factors.

During OPC differentiation into mature OLs cells undergo major changes in their protein and lipid metabolism including expression levels of hormone receptors with a different responsiveness to PDGF and FGF-2 [40]. Isolated cells of the OL-lineage were treated with a combination of PDGF and FGF-2 to differentiate between IgM-mediated effects on OPCs compared to mature OLs. FGF-2 enhances PDGF-stimulated OPC proliferation by inducing PDGFαR expression and inhibition of OPC differentiation [41]–[44]. A previous study demonstrated IgM-mediated OPC apoptotic signaling and differentiation to occur days after IgM administration [24]. However, PDGF alone is not equally sufficient compared to the combination of PDGF with FGF-2 to keep OL-lineage cells for days in a proliferative OPC stage. Results on IgM-stimulated OPC proliferation in mixed glia strongly suggest the PDGF pathway to transduce effects by rHIgM22 into OPCs, which is confirmed in isolated OPCs. Due to the combined effect of rHIgM22 with PDGF and FGF-2 in isolated OPC culture, however, it appears to be likely that FGF-2 enhances rHIgM22-mediated effects on OPCs as well either in combination with PDGF or potentially alone. Alternatively, FGF-2 may potentially enhance rHIgM22-mediated Lyn expression and inhibition of OPC apoptotic signaling by keeping cells in an immature, proliferative OPC stage with higher PDGFαR and Lyn levels compared to mature OLs.

The SFK Lyn is involved in PDGF-mediated proliferation and survival of OPCs in an integrin αvβ3-dependent manner [25], [27]. Induction of Lyn expression is the most stable response to rHIgM22 in isolated OPCs. The induction and activation of Lyn kinase inhibits apoptosis in OPCs [24] and may also inhibit OPC differentiation. PDGF and FGF-2, on the other hand, induce Lyn expression, which is strongly enhanced by adding rHIgM22 to OPC cultures, demonstrating a synergistic effect between rHIgM22 and PDGF/FGF-2. This suggests that rHIgM22 may require high levels of PDGF secreted by astrocytes with Lyn as a potential intracellular mediator for remyelination. In the absence of PDGF and FGF-2, OPCs differentiate rapidly into mature OLs. The rHIgM22-mediated induction of Lyn expression is constant over 7 days in the presence of PDGF and FGF-2 but continuously decreases over time in the absence of both growth factors. This demonstrates that rHIgM22 can induce Lyn expression without PDGF and FGF-2. The gradual decline in Lyn expression with rHIgM22-treatment likely reflects fewer cells responding to rHIgM22, since mature MBP-positive OLs present at day 7 in culture do not respond to rHIgM22 treatment. This suggests OPCs but not mature MBP-positive OLs respond to rHIgM22. Terminally differentiated OLs down-regulate many cell surface receptors and intracellular signaling molecules, including endogenous Lyn levels, which may constrain the response to rHIgM22.

OPC proliferation is intrinsically tied to OL differentiation. Maintaining OPCs in a proliferative state inhibits their differentiation into myelinating OLs [41], [92]. Lowering the growth factor threshold required to drive OPC proliferation may explain how rHIgM22 inhibits OPC differentiation. This reinforces the observations of other investigators and demonstrates that polyclonal IgM preparations (IVIgMs) and the IgM O4 inhibit OPC differentiation *in vitro* [22], [23]. Of note, both reagents also promote remyelination *in vivo* [18], [21]. Blocked OPC differentiation following rHIgM22 treatment may correlate to decreased apoptosis. OPC maturation *in vitro* is linked to a substantial amount of cell death, and stalling the differentiation process results in less apoptosis. There is no evidence that rHIgM22 reverses the maturation of MBP-positive OLs into OPCs: mature OL cultures grown without PDGF and FGF-2 do not respond to rHIgM22.

Similar to the process demonstrated with the IgM O4, rHIgM22-mediated inhibition of OPC differentiation may be reversible [23]. The half-life of rHIgM22 in mice is 15 h, and serum rHIgM22-concentrations are close to zero 48 h after administration [19]. This suggests that normal OPC differentiation proceeds after a burst of IgM driven proliferation. The inflammatory milieu of a lesion may keep OPCs quiescent until a supportive environment evolves to support remyelination [22].

In summary, this is the first study demonstrating rHIgM22-mediated OPC proliferation. We observed that the PDGF-pathway is necessary, but not sufficient, for rHIgM22-stimulated OPC proliferation and survival. Our data support the hypothesis that promoting OPC proliferation and survival, not OPC differentiation into mature OLs or survival of mature OLs, are the primary mediators of IgM remyelination. We propose that remyelinating IgMs *in vivo* lower the OPC-response threshold to endogenous soluble factors. IgMs at the lesion site allow OPCs to respond naturally to an environment that does not adequately support remyelination.

## Supporting Information

Figure S1 **rHIgM22 stimulates proliferation of OPCs but not astrocytes or microglia in mixed glial cultures.** Mixed glial cells were maintained in serum-containing medium for 5 days prior to the addition of rHIgM22 or isotype-control IgM (IC) (10 µg/ml each) in serum-free media for 48 h. Representative double immunofluorescence images showing proliferation marker Ki-67 with either Olig-1, Olig-2, GFAP or CD68 including DAPI-staining for both treatment groups (rHIgM22 vs IC).
(TIF)

Figure S2 **rHIgM22 stimulates expression of OPC markers in mixed glial cultures but not in isolated OPCs.** Mixed glial cells were maintained in serum-containing medium for 5 days prior to the addition of rHIgM22 or isotype-control IgM (IC) (10 µg/ml each) or PDGF and FGF-2 (10 ng/ml each) in serum-free media for 48 h. **A.** Representative Western blots from one of three independent experiments in mixed glial cultures showing levels of Olig-1, Olig-2, GFAP, CD68 and β-actin as a loading control. **B.** Quantitative analysis of Western blots from 3 independent experiments as described under A. Background is subtracted from each value and normalized against β-actin. Data are presented as mean ± S.D. (*n = *3). * *p*<0.05 compared to controls. **C.** Representative Western blots from one of three independent experiments of isolated OPCs treated for 1–7 days with rHIgM22 or human isotype control IgM (10 µg/ml each) in the absence of PDGF/FGF-2 showing levels of OPC markers NG2, PDGFαR, Olig-2 in addition to loading controls β-actin and histone H3.
(TIF)

Figure S3 **rHIgM22-mediated activation of Lyn, ERK1 and ERK2 requires PDGF and FGF-2.** Quantitative analysis of Western blots from 3 independent experiments in isolated OL cultures grown on fibronectin and treated for 1–7 days with isotype- control IgM (IC) or rHIgM22 (10 µg/ml each) in the absence (**Figure S3**) or presence (**Figure S4**) of PDGF/FGF-2 (10 ng/ml each). Background is subtracted from each value and normalized against β-actin. Data are presented as mean ± S.D. (*n = *3). * *p*<0.05 compared to controls.
(TIF)

Figure S4 **rHIgM22-mediated activation of Lyn, ERK1 and ERK2 requires PDGF and FGF-2.** Quantitative analysis of Western blots from 3 independent experiments in isolated OL cultures grown on fibronectin and treated for 1–7 days with isotype- control IgM (IC) or rHIgM22 (10 µg/ml each) in the absence (**Figure S3**) or presence (**Figure S4**) of PDGF/FGF-2 (10 ng/ml each). Background is subtracted from each value and normalized against β-actin. Data are presented as mean ± S.D. (*n = *3). * *p*<0.05 compared to controls.
(TIF)

Figure S5 **PDGF is necessary but not sufficient for rHIgM22-stimulated proliferation in isolated OPCs.** Isolated OPCs were maintained for 24 h after plating in serum-containing (1.5%) medium. After switching to serum-free media OPCs were incubated for 48 h with rHIgM22 or isotype-control IgM (IC) (10 µg/ml each) plus PDGF and FGF-2 (10 ng/ml each). BRDU (10 µM final concentration) was added into the medium for 18 h. Fixed and permeabilized cells were stained with anti-BRDU antibody for 4 h and processed for immunocytochemistry. **A.** Representative images (60×) of anti- BRDU, DAPI and phase contrast of OPC cultures treated with rHIgM22, IC and medium only. **B.** Quantitative analysis of BRDU-positive cells per microscopic 10× view field with >10.000 counted cells in each treatment group. **C+D.** Qualitative and quantitative analysis of OPC cultures described under A and B. **C.** Representative immunofluorescence images show labeling of OPC cultures with A2B5, O4, O1 (cells of the OL-lineage), CD68 (microglia, macrophages) and GFAP (astrocytes) plus DAPI. **D.** Quantitative analysis of immunofluorescence images (10× view field) with >10.000 counted cells and normalized to the number of DAPI-positive cells from the same images.
(TIF)

Table S1 **IgM and growth factor mediated tritium uptake into mixed glia**
(DOCX)
