# Supplementary figures and images for: PDGF is Required for Remyelination-Promoting IgM Stimulation of Oligodendrocyte Progenitor Cell Proliferation
Source: PLoS One. 2013 Feb 1;8(2):e55149. doi: 10.1371/journal.pone.0055149 (PMC3562326; doi:10.1371/journal.pone.0055149)

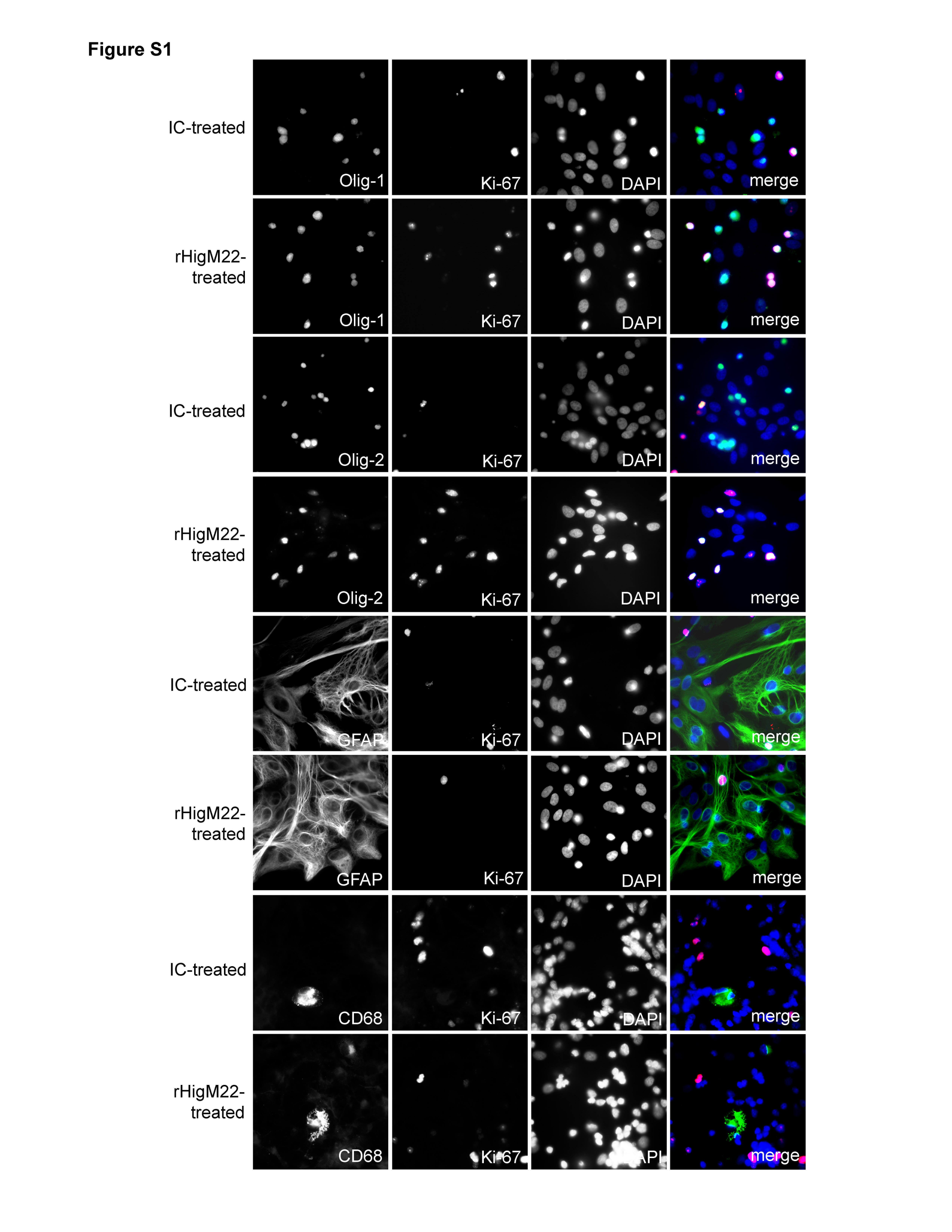

Supplement: Figure S1 — rHIgM22 stimulates proliferation of OPCs but not astrocytes or microglia in mixed glial cultures. Mixed glial cells were maintained in serum-containing medium for 5 days prior to the addition of rHIgM22 or isotype-control IgM (IC) (10 µg/ml each) in serum-free media for 48 h. Representative double immunofluorescence images showing proliferation marker Ki-67 with either Olig-1, Olig-2, GFAP or CD68 including DAPI-staining for both treatment groups (rHIgM22 vs IC). (TIF) [file pone.0055149.s001.tif]

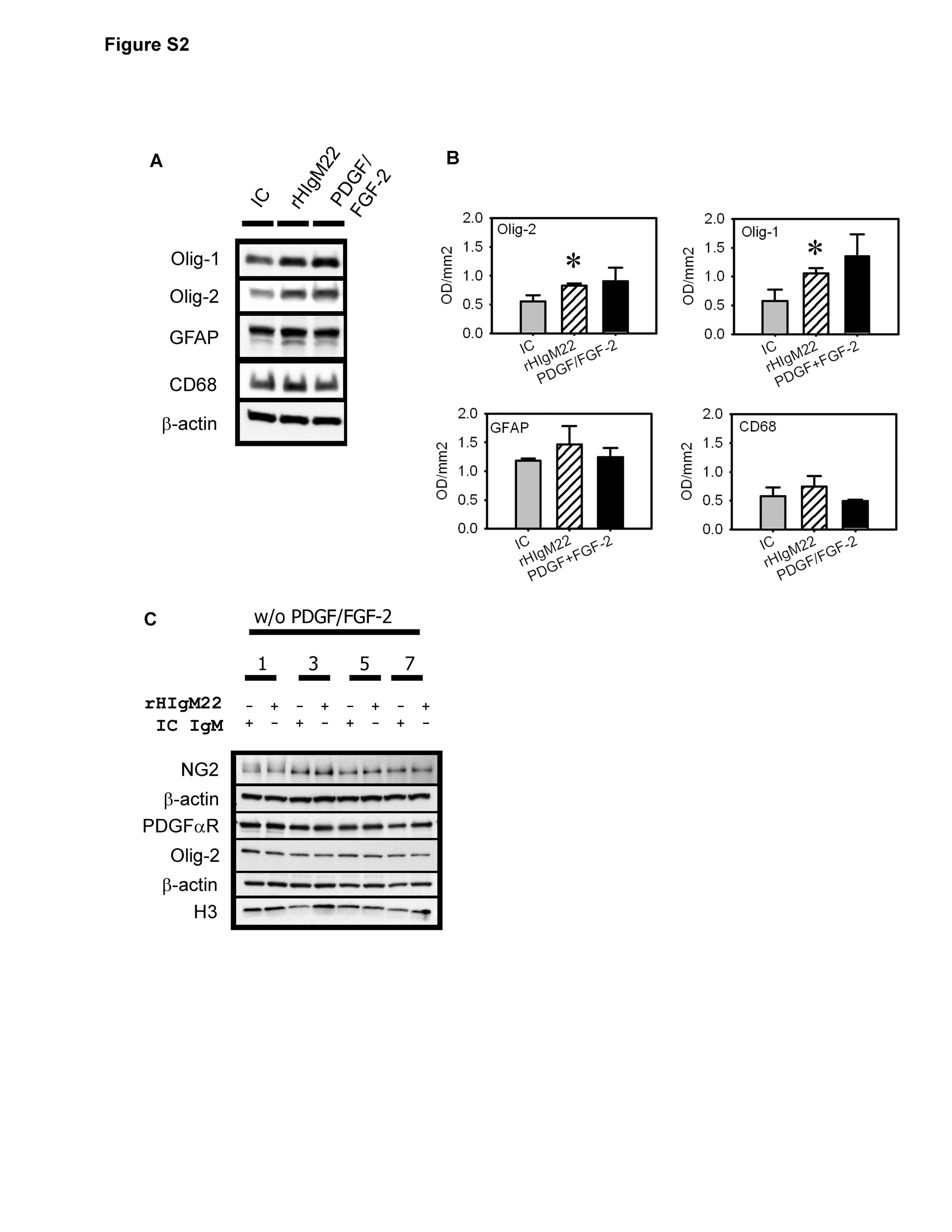

Supplement: Figure S2 — rHIgM22 stimulates expression of OPC markers in mixed glial cultures but not in isolated OPCs. Mixed glial cells were maintained in serum-containing medium for 5 days prior to the addition of rHIgM22 or isotype-control IgM (IC) (10 µg/ml each) or PDGF and FGF-2 (10 ng/ml each) in serum-free media for 48 h. A. Representative Western blots from one of three independent experiments in mixed glial cultures showing levels of Olig-1, Olig-2, GFAP, CD68 and β-actin as a loading control. B. Quantitative analysis of Western blots from 3 independent experiments as described under A. Background is subtracted from each value and normalized against β-actin. Data are presented as mean ± S.D. (n = 3). * p<0.05 compared to controls. C. Representative Western blots from one of three independent experiments of isolated OPCs treated for 1–7 days with rHIgM22 or human isotype control IgM (10 µg/ml each) in the absence of PDGF/FGF-2 showing levels of OPC markers NG2, PDGFαR, Olig-2 in addition to loading controls β-actin and histone H3. (TIF) [file pone.0055149.s002.tif]

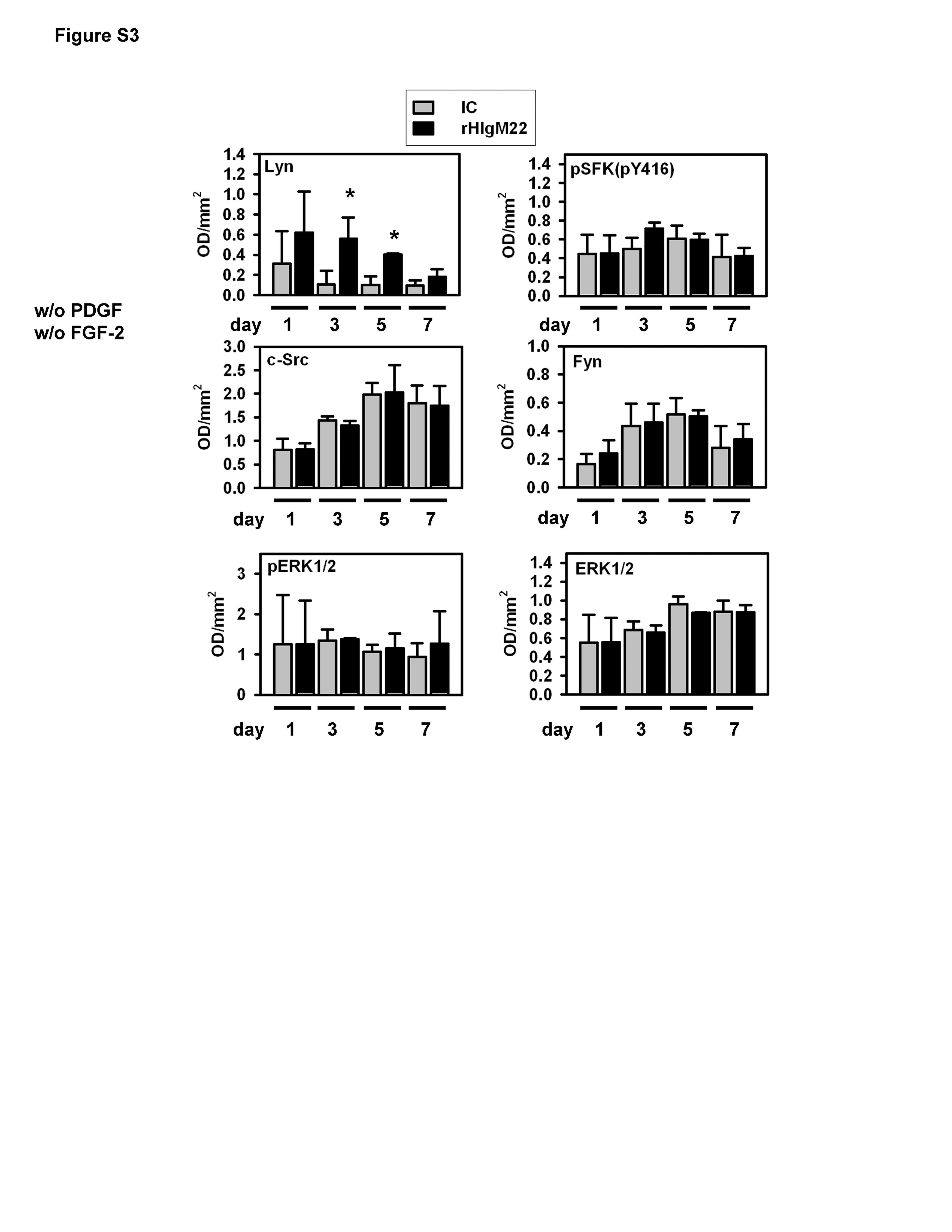

Supplement: Figure S3 — rHIgM22-mediated activation of Lyn, ERK1 and ERK2 requires PDGF and FGF-2. Quantitative analysis of Western blots from 3 independent experiments in isolated OL cultures grown on fibronectin and treated for 1–7 days with isotype- control IgM (IC) or rHIgM22 (10 µg/ml each) in the absence (Figure S3) or presence (Figure S4) of PDGF/FGF-2 (10 ng/ml each). Background is subtracted from each value and normalized against β-actin. Data are presented as mean ± S.D. (n = 3). * p<0.05 compared to controls. (TIF) [file pone.0055149.s003.tif]

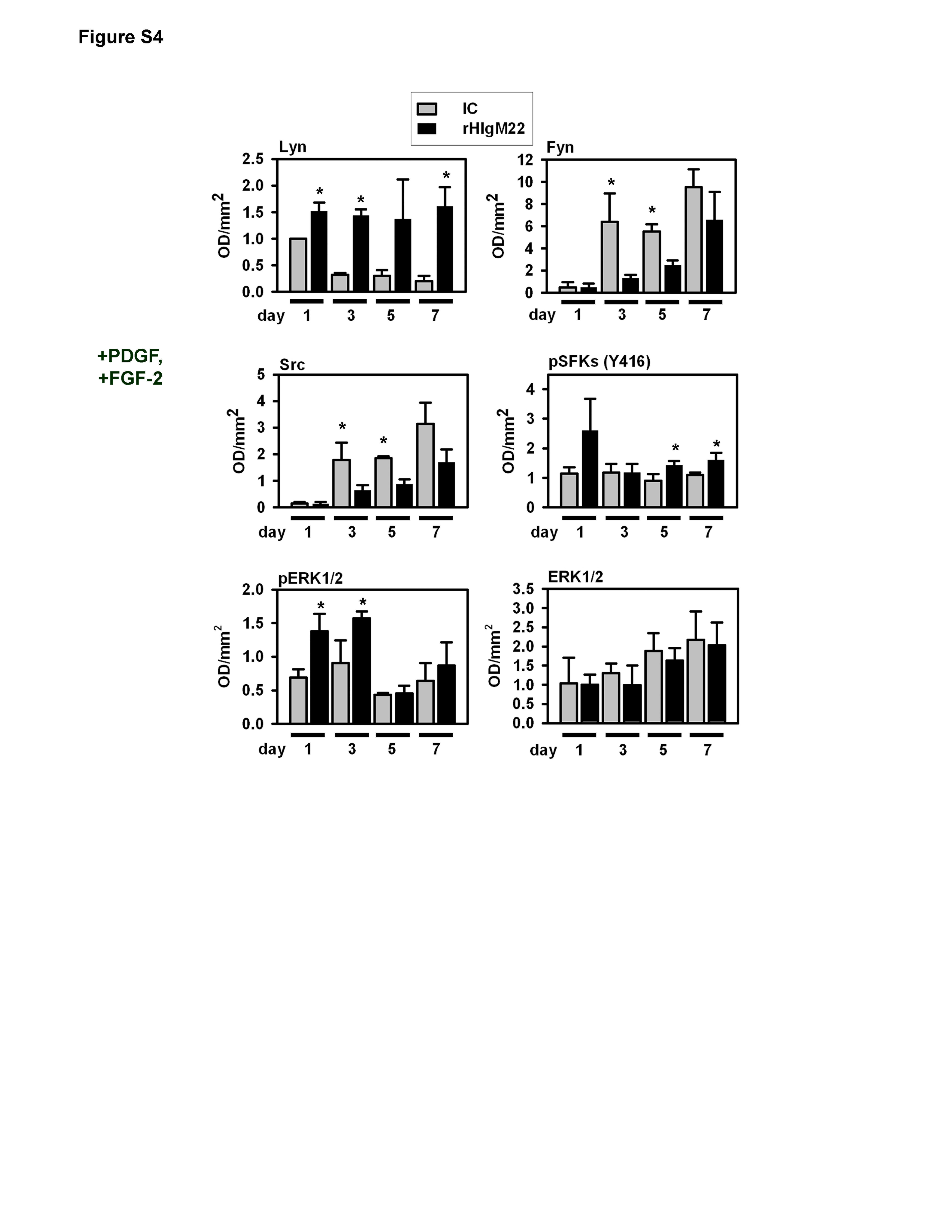

Supplement: Figure S4 — rHIgM22-mediated activation of Lyn, ERK1 and ERK2 requires PDGF and FGF-2. Quantitative analysis of Western blots from 3 independent experiments in isolated OL cultures grown on fibronectin and treated for 1–7 days with isotype- control IgM (IC) or rHIgM22 (10 µg/ml each) in the absence (Figure S3) or presence (Figure S4) of PDGF/FGF-2 (10 ng/ml each). Background is subtracted from each value and normalized against β-actin. Data are presented as mean ± S.D. (n = 3). * p<0.05 compared to controls. (TIF) [file pone.0055149.s004.tif]

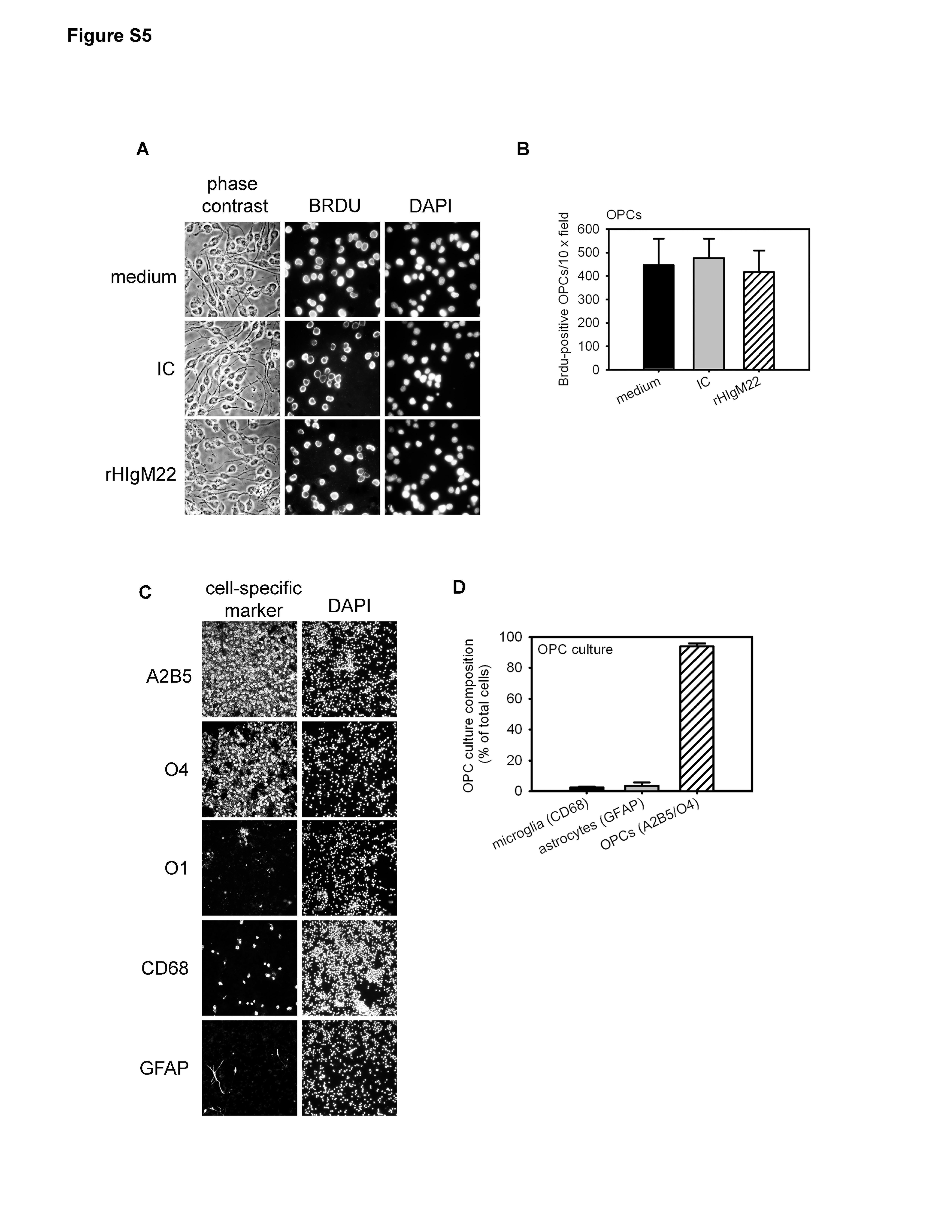

Supplement: Figure S5 — PDGF is necessary but not sufficient for rHIgM22-stimulated proliferation in isolated OPCs. Isolated OPCs were maintained for 24 h after plating in serum-containing (1.5%) medium. After switching to serum-free media OPCs were incubated for 48 h with rHIgM22 or isotype-control IgM (IC) (10 µg/ml each) plus PDGF and FGF-2 (10 ng/ml each). BRDU (10 µM final concentration) was added into the medium for 18 h. Fixed and permeabilized cells were stained with anti-BRDU antibody for 4 h and processed for immunocytochemistry. A. Representative images (60×) of anti- BRDU, DAPI and phase contrast of OPC cultures treated with rHIgM22, IC and medium only. B. Quantitative analysis of BRDU-positive cells per microscopic 10× view field with >10.000 counted cells in each treatment group. C+D. Qualitative and quantitative analysis of OPC cultures described under A and B. C. Representative immunofluorescence images show labeling of OPC cultures with A2B5, O4, O1 (cells of the OL-lineage), CD68 (microglia, macrophages) and GFAP (astrocytes) plus DAPI. D. Quantitative analysis of immunofluorescence images (10× view field) with >10.000 counted cells and normalized to the number of DAPI-positive cells from the same images. (TIF) [file pone.0055149.s005.tif]
