# Supplementary material for: PDGF is Required for Remyelination-Promoting IgM Stimulation of Oligodendrocyte Progenitor Cell Proliferation
Source: PLoS One. 2013 Feb 1;8(2):e55149. doi: 10.1371/journal.pone.0055149 (PMC3562326; doi:10.1371/journal.pone.0055149)
Supplement: Table S1 — IgM and growth factor mediated tritium uptake into mixed glia (DOCX) [file pone.0055149.s006.docx]

**Table S1**

|  | concentration | Tritium counts |
| --- | --- | --- |
| PDGF/FGF-2 | 0.2 ng/ml PDGF; 0.1 ng/ml FGF-2 | 10787 ± 233 |
| PDGF/FGF-2 | 2 ng/ml PDGF; 1 ng/ml FGF-2 | 13277 ± 447 |
| PDGF/FGF-2 | 20 ng/ml PDGF; 10 ng/ml FGF-2 | 24245 ± 355 |
| PDGF/FGF-2 | 200 ng/ml PDGF; 100 ng/ml FGF-2 | 32248 ± 596 |
| rHIgM22 | 0.1 μg/ml | 12023 ± 472 |
| rHIgM22 | 1 μg/ml | 16571 ± 410 |
| rHIgM22 | 10 μg/ml | 20367 ± 621 |
| rHIgM22 | 20 μg/ml | 24687 ± 755 |
| isotype control IgM | 0.1 μg/ml | 11116 ± 634 |
| isotype control IgM | 1 μg/ml | 10129 ± 783 |
| isotype control IgM | 10 μg/ml | 12855 ± 832 |
| isotype control IgM | 20 μg/ml | 12740 ± 2446 |
| media |  | 11391 ± 985 |
| media |  | 11243 ± 719 |
| media |  | 11008 ± 886 |
| media |  | 13224 ± 950 |
